# Supplementary material for: Preferences for long-acting injectable HIV pre-exposure prophylaxis service delivery among male and female sex workers in Uganda: A discrete choice experiment
Source: PLOS Glob Public Health. 2025 Oct 9;5(10):e0004798. doi: 10.1371/journal.pgph.0004798 (PMC12510523; doi:10.1371/journal.pgph.0004798)
Supplement: S1 Text — (DOCX) [file pgph.0004798.s001.docx]

MSW and FSW survey questionnaire on PrEP service delivery attributes, preferred delivery models, willingness to pay among MSW and FSW- version 1.0

**SECTION A: SOCIODEMOGRAPHIC CHARACTERISTICS**

| No. | Questions and filters | Answers and codes | Shift to |
| --- | --- | --- | --- |
| Q01 | Location | Mbarara……………………1  Kampala……………………2 |  |
| Q02 | Sex | Male………………………….1  Female…………………………2 |  |
| Q03 | Age in complete years |  |  |
| Q04 | Highest level of education attained; primary, secondary, higher education  *Interviewer, circle only one answer* | No education…………..0  Primary …………………….1  Secondary……………………2  Higher education……………..3 |  |
| Q05 | Marital status | Married………………….1  Widow…………………….2  Single………………………3  Regular boy/girl friend……….4 |  |
| Q05 | Number (biological) of children currently |  |  |
| Q06 | Duration in sex work in completed months |  |  |
| Q07 | How would describe your current work  *Interviewer, list all the possibilities* | Full time, as I have no other sources of income……………………….1  Part-time, as I have other sources of income……………………….2  Part-time, as I am a student……...3  Full-time, to supplement my other sources of income……………………………….4  Others specify………………………. |  |
| Q08 | Client Solicitation strategy  *Interviewer, list all the possibilities. There can be more than one answer circled* | Street ……………………………………..1  Home………………………………………2  Lodge……………………………………….3  Bar…………………………………………..4  Brothel………………………………………5  Website……………………………………..6  Social media……………………………….7  Procurer…………………………………….8  Others specific………………………………. |  |
| 09 | Average number of clients per week |  |  |
| 10 | Average amount of money earned per week in Uganda shillings |  |  |

**SECTION B: Condom use and STI/HIV testing behaviors**

| No. | Questions and filters | Answers and codes | Shift to |
| --- | --- | --- | --- |
| Q01 | Used condom at last sex | No …………..0  Yes …………………….1 |  |
| Q02 | Description of condom use | consistently use at all times with all clients ………….1 sometimes nonuse with regular clients…………….2  sometimes nonuse if the client requests so……………3  Sometimes nonuse for more money………………4 sometimes nonuse if I like the client by look ………5  Sometimes nonuse if we test for HIV …………….6  Others specify…………………….. |  |
| Q03 | Syphilis testing frequency in the prior 6 months | 0…………………………1  1…………………………2  ≥2……………………….3 |  |
| Q04 | HIV testing frequency in the prior 6 months | 0…………………………1  1…………………………2  ≥2……………………….3 |  |
| Q05 | HIV testing modality if done in the prior 6 months | Self-testing………………1  Visited a health facility…….2  Outreach…………………….3  Others specify ………………. |  |
| Q06 | Self-rating of intention to test for HIV in the next 3 months | No intention…………………1  Low intention……………….2  High intention………………3  Not sure………………….4 |  |
| Q06 | Self-rating of intention to take a serological syphilis test in the next 3 months | No intention…………………1  Low intention……………….2  High intention………………3  Not sure………………….4 |  |

**SECTION C: HIV risk perception**

| No. | Questions and filters | Answers and codes | Shift to |
| --- | --- | --- | --- |
| Q1 | How serious is contracting HIV as a health problem | Not very serious…………..1  Not serious…………………2  Slightly serious…………….3  Neither serious nor very serious……………………..4  Serious …………………….5  Very serious……………….. |  |
| Q2 | Current level of risk of being infected with HIV if not already infected | Very low……………….1  Low…………………….2  Slightly low…………….3  Neither low nor high……4  High……………………..5  Very high………………...6 |  |
| Q3 | Reasons you evaluate your current level of risk that way.  *Interviewer, many possible answers* |  |  |

**SECTION D: PrEP awareness and use**

| No. | Questions and filters | Answers and codes |  |
| --- | --- | --- | --- |
| Q1 | Aware about PrEP for HIV prevention | No……………….1  Yes………………2 | 1 10 |
| Q2 | If yes, what was the source of your information | HCW………………1  Peers……………….2  Newspaper…………3  Radio ………………4  Others specify…………………. |  |
| Q3 | If already aware, which PrEP options are you aware of  *Interviewer, many possible answers, circle all mentioned* | Oral …………1  Vaginal ring-----------2  Injectable ……………3 |  |
| Q4 | Regarding oral PrEP, which modalities are you aware of  *Interviewer, many possible answers* | Daily oral ………….1  Event-driven/on-demand……….2  I don’t know………………3 |  |
| Q5 | You are aware of where to obtain PrEP services | No…………….1  Yes……………..2 |  |
| Q6 | Oral PrEP use | Never used……….1  Ever used but not currently using……………2  Currently using………………………...3 |  |
| Q7 | Reasons for never using | …………………………………………………………………  ………………………………………………………………..  ………………………………………………………………  …………………………………………………………….. |  |
| Q9 | Reasons for noncurrent use | ………………………………………………………………..  ………………………………………………………………..  ………………………………………………………………  ……………………………………………………………… |  |
| Q10 | PrEP is a drug given to someone who is HIV negative to reduce their chance of acquiring HIV. How would you rate your willingness to use PrEP for HIV prevention | Very low…………………………1  Low ……………………………...2  High …………………………….3  Very high……………………….4 |  |
| Q11 | PrEP exists in different modalities. Among these options, which is your preferred choice | Oral daily PrEP (oral tablet swallowed daily) ………….1  Event-driven/on-demand PrEP (oral tablets swallowed 24 hrs before sex) ………………………………………………2  Injectable PrEP given as an injection every 2 months…….3  Vaginal ring (only for women and inserted 24 hours in the vagina before sex) ………………………………………4 |  |
| Q12 | If injectable PrEP becomes available at nominal fee, would you be willing to pay for it | Not willing at all…………….1  Willing depending on the cost…….2  Maybe………………………………3 |  |
| Q13 | If you are willing to pay for the injection, what would be the maximum amount in Ugandan shillings you are willing to pay for it |  |  |

**Section E: PrEP service delivery attributes & preferred delivery models (DCE)**

Government of Uganda is planning to roll out Injectable PrEP for HIV prevention. Individuals choosing to use this method will receive an injection every 2 months. You are provided with 10 choice cards containing

different hypothetical scenarios of how this service could be delivered. Each choice has four choice options with an opt-out option. You are requested to choose only one row per choice card of that best represents how you would prefer to seek injectable PrEP every 2 months when available.

**Choice card ^1**

| Card choice | **location** | **Waiting time** | **Provider** | **Additional services** | selected choice |
| --- | --- | --- | --- | --- | --- |
| 1 | Injectable PrEP is provided by government hospital, you travel to the Family planning clinic | You wait 2 hours to be seen by HCW | You meet a female HCW | In addition to receiving PrEP injection, you checked for diabetes and hypertension |  |
| 1 | Injectable PrEP is provided by a private clinic | You wait < 1 hour to be seen by HCW | You meet a male HCW | In addition to receiving PrEP injection, you are checked for cancer i.e. cervical or prostate |  |
| 1 | Injectable PrEP is provided by government hospital, you travel to the ART clinic | You wait 1 hour to be seen by HCW | You meet a female peer | In addition to receiving PrEP injection, you receive pyschosocial support |  |
| 1 | Injectable PrEP is provided by NGO-run mobile outreach, you attend the outreach in your community | You wait 1 hour to be seen by HCW | You meet a male peer | In addition to receiving PrEP injection, you receive risk counseling |  |
| 1 | Opt-out | Opt-out | Opt-out | Opt-out |  |

**Choice card ^2**

|  | **location** | **Waiting time** | **Provider** | **Additional services** | selected choice |
| --- | --- | --- | --- | --- | --- |
| 2 | Injectable PrEP is provided a private pharmacy | You wait 2 hours to be seen by HCW | You meet a female peer | In addition to receiving PrEP injection, you receive contraceptives/condoms |  |
| 2 | Injectable PrEP is provided by government hospital, you travel to the Family planning clinic | You wait 1 hour to be seen by HCW | You meet a male HCW | In addition to receiving PrEP injection, you receive pyschosocial support |  |
| 2 | Injectable PrEP is provided by government hospital, you travel to the district hospital | You wait <1 hour to be seen by HCW | You meet a male peer | In addition to receiving PrEP injection, you are checked for diabetes and hypertension |  |
| 2 | Injectable PrEP is provided by government hospital, you travel to the STI clinic | You wait <1 hour to be seen by HCW | You meet a male peer | In addition to receiving PrEP injection, you receive risk counseling |  |
| 2 | Opt-out | Opt-out | Opt-out | Opt-out |  |

**Choice card ^3**

|  | **location** | **Waiting time** | **Provider** | **Additional services** | selected choice |
| --- | --- | --- | --- | --- | --- |
| 3 | Injectable PrEP is provided by government hospital, you travel to the Family planning clinic | You wait < 1 hour to be seen by HCW | You meet a female peer | In addition to receiving PrEP injection, you receive risk counseling |  |
| 3 | Injectable PrEP is provided by government hospital, you travel to the community health center | You wait 1 hour to be seen by HCW | You meet a female HCW | In addition to receiving PrEP injection, you are checked for diabetes and hypertension |  |
| 3 | Injectable PrEP is provided by  NGO-run drop-in center  you travel to the center in your community | You wait 2 hours to be seen by HCW | You meet a male HCW | In addition to receiving PrEP injection, you are checked for cancer i.e. cervical or prostate |  |
| 3 | Injectable PrEP is provided by NGO-run mobile outreach, you attend the outreach in your community | You wait 2 hours to be seen by HCW | You meet a male HCW | In addition to receiving PrEP injection, pyschosical support |  |
| 3 | Opt-out | Opt-out | Opt-out | Opt-out |  |

**Choice card ^4**

|  | **location** | **Waiting time** | **Provider** | **Additional services** | selected choice |
| --- | --- | --- | --- | --- | --- |
| 4 | Injectable PrEP is provided by MARPI, you travel to the MARPI clinic | You wait 2 hours to be seen by HCW | You meet a female HCW | In addition to receiving PrEP injection, you receive risk counseling |  |
| 4 | Injectable PrEP is provided by an NGO-run drop-in center  , you travel to the drop-in center in your community | You wait < 1 hour to be seen by HCW | You meet a female HCW | In addition to receiving PrEP injection, you receive pyschosocial support |  |
| 4 | Injectable PrEP is provided by  A government hospital, you travel to the family planning clinic | You wait 1 hours to be seen by HCW | You meet a male peer | In addition to receiving PrEP injection, you are checked for cancer i.e. cervical or prostate |  |
| 4 | Injectable PrEP is provided by a government hospital, you travel to an STI clinic | You wait 2 hours to be seen by HCW | You meet a male HCW | In addition to receiving PrEP injection, your receive contraceptives/condoms |  |
| 4 | Opt-out | Opt-out | Opt-out | Opt-out |  |

**Choice card ^5**

|  | **location** | **Waiting time** | **Provider** | **Additional services** | selected choice |
| --- | --- | --- | --- | --- | --- |
| 5 | Injectable PrEP is provided by a private clinic, you travel to a private clinic in your community | You wait 2 hours to be seen by HCW | You meet a male peer | In addition to receiving PrEP injection, you are checked for diabetes and hypertension |  |
| 5 | Injectable PrEP is provided by government hospital, you travel to the district hospital in your community | You wait 1 hour to be seen by HCW | You meet a female peer | In addition to receiving PrEP injection, you are checked for cancer i.e. cervical or prostate |  |
| 5 | Injectable PrEP is provided by  a government hospital, you travel to the STI clinic | You wait < 1 hour to be seen by HCW | You meet a female HCW | In addition to receiving PrEP injection, you receive pyschoscial support |  |
| 5 | Injectable PrEP is provided by a government health center, you travel to the health center in your community | You wait < 1 hour to be seen by HCW | You meet a male HCW | In addition to receiving PrEP injection, your receive contraceptives/condoms |  |
| 5 | Opt-out | Opt-out | Opt-out | Opt-out |  |

**Choice card ^6**

|  | **location** | **Waiting time** | **Provider** | **Additional services** | selected choice |
| --- | --- | --- | --- | --- | --- |
| 6 | Injectable PrEP is provided by a private pharmacy, you travel to a private in your community | You wait < 1 hour to be seen by HCW | You meet a male peer | In addition to receiving PrEP injection, you receive pyschosocial support |  |
| 6 | Injectable PrEP is provided by government hospital, you travel to the ART clinic | You wait <1 hour to be seen by HCW | You meet a female HCW | In addition to receiving PrEP injection, you are checked for diabetes and hypertension |  |
| 6 | Injectable PrEP is provided by a private clinic, you travel to a private clinic in your community | You wait 1 hour to be seen by HCW | You meet a female HCW | In addition to receiving PrEP injection, you receve contraceptives/condoms |  |
| 6 | Injectable PrEP is provided by MARPI, you travel to the MARPI clinic | You wait 2 hours to be seen by HCW | You meet a female peer | In addition to receiving PrEP injection, you are checked for caner i.e cervical and prostate |  |
| 6 | Opt-out | Opt-out | Opt-out | Opt-out |  |

**Choice card ^7**

|  | **location** | **Waiting time** | **Provider** | **Additional services** | selected choice |
| --- | --- | --- | --- | --- | --- |
| 7 | Injectable PrEP is provided by government hospital, you travel to the ART clinic | You wait 2 hours to be seen by HCW | You meet a male peer | In addition to receiving PrEP injection, you receive contraceptives/condoms |  |
| 7 | Injectable PrEP is provided by  NGO-run mobile outreach, you attend the outreach in your community | You wait <1 hour to be seen by HCW | You meet a female HCW | In addition to receiving PrEP injection, you are checked for cancer i.e. cervical or prostate |  |
| 7 | Injectable PrEP is provided by a government health center, you travel to the health center in your community | You wait 2 hours to be seen by HCW | You meet a female peer | In addition to receiving PrEP injection, you receve risk counseling |  |
| 7 | Injectable PrEP is provided by a government hospital, you travel to the STI clinic | You wait 1 hours to be seen by HCW | You meet a male HCW | In addition to receiving PrEP injection, you are checked for diabetes and hypertension |  |
| 7 | Opt-out | Opt-out | Opt-out | Opt-out |  |

**Choice card ^8**

|  | **location** | **Waiting time** | **Provider** | **Additional services** | selected choice |
| --- | --- | --- | --- | --- | --- |
| 8 | Injectable PrEP is provided by government hospital, you travel to the district hospital | You wait 2 hours to be seen by HCW | You meet a female HCW | In addition to receiving PrEP injection, you receive pyschosocial support |  |
| 8 | Injectable PrEP is provided by a government hospital, you travel to the ART clinic | You wait 1 hour to be seen by HCW | You meet a male HCW | In addition to receiving PrEP injection, you receive risk counseling |  |
| 8 | Injectable PrEP is provided at MARPI clinic | You wait < 1 hour to be seen by HCW | You meet a male peer | In addition to receiving PrEP injection, you receive contraceptives/condoms |  |
| 8 | Injectable PrEP is provided by an  NGO-run mobile outreach, you attend the outreach in your community | You wait < 1 hour to be seen by HCW | You meet a female peer | In addition to receiving PrEP injection, you are checked for diabetes and hypertension |  |
| 8 | Opt-out | Opt-out | Opt-out | Opt-out |  |

**Choice card ^9**

|  | **location** | **Waiting time** | **Provider** | **Additional services** | selected choice |
| --- | --- | --- | --- | --- | --- |
| 9 | Injectable PrEP is provided by government health center, you travel to the health center in your community | You wait 2 hours to be seen by HCW | You meet a male peer | In addition to receiving PrEP injection, you are checked for cancer i.e. cervical or prostate |  |
| 9 | Injectable PrEP is provided at MARPI clinic | You wait 1 hour to be seen by HCW | You meet a female peer | In addition to receiving PrEP injection, you are checked for diabetes and hypertension |  |
| 9 | Injectable PrEP is provided by an  NGO-run drop-in center, you travel to the center in your community | You wait 2 hours to be seen by HCW | You meet a female HCW | In addition to receiving PrEP injection, you receive risk counseling |  |
| 9 | Injectable PrEP is provided by a government hospital, you travel to the district hospital in your community | You wait < 1 hour to be seen by HCW | You meet a male HCW | In addition to receiving PrEP injection, you receive contraceptives/condoms |  |
| 9 | Opt-out | Opt-out | Opt-out | Opt-out |  |

**Choice card ^10**

|  | **location** | **Waiting time** | **Provider** | **Additional services** | selected choice |
| --- | --- | --- | --- | --- | --- |
| 10 | Injectable PrEP is provided by an  NGO-run drop-in center, you travel to the center in your community | You wait 1 hour to be seen by HCW | You meet a male peer | In addition to receiving PrEP injection, you receive contraceptives/condoms |  |
| 10 | Injectable PrEP is provided by a private clinic, you travel to a private clinic in your community | You wait < 1 hour to be seen by HCW | You meet a female peer | In addition to receiving PrEP injection, you receive pyschosocial support |  |
| 10 | Injectable PrEP is provided by a private drugs shop, you travel to a private drugs shop in your community | You wait 2 hours to be seen by HCW | You meet a male HCW | In addition to receiving PrEP injection, you are checked for diabetes and hypertension |  |
| 10 | Injectable PrEP is provided by a private pharmacy, you travel to the private pharmacy in your community | You wait 1 hour to be seen by HCW | You meet a female HCW | In addition to receiving PrEP injection, you checked for cancer i.e. cervical or prostate |  |
| 10 | Opt-out | Opt-out | Opt-out | Opt-out |  |
