# Supplementary material for: Preferences for long-acting injectable HIV pre-exposure prophylaxis service delivery among male and female sex workers in Uganda: A discrete choice experiment
Source: PLOS Glob Public Health. 2025 Oct 9;5(10):e0004798. doi: 10.1371/journal.pgph.0004798 (PMC12510523; doi:10.1371/journal.pgph.0004798)
Supplement: Attachment — Submitted filename: Response to Reviewers_clean.doc [file pgph.0004798.s005.doc]

**Makerere	University**

P.O. Box 7072 Kampala, Uganda	**Tel:** 256 414 530404

**E-mail**: [nursing@chs.mak.ac.ug](mailto:nursing@chs.mak.ac.ug)

**SCHOOL OF HEALTH SCIENCES**

**Department of Nursing**

Dr. Emma Campbell

Staff Editor, Plos Global Public Health

July 26^th^, 2025

Dear Dr. Campbell,

Regarding PGPH-D-25-01202 - EMID:139ff34e13bdc6c1

We are very grateful for the reviewer comments provided by the reviewers of our manuscript: “Associations with HIV Preexposure Prophylaxis Use by Cisgender Female Sex Workers in Two Ugandan Cities ". Reviewer comments are encouraging, and the reviewers appear to share our view that study findings are of public health importance. The suggestions offered by the reviewers have been greatly helpful. Please find below our point-by-point responses in italics, with new text in bold font:

**Reviewer Comments**

Reviewer #1:

1. This is a good paper that brings out a critical component of preferences by location, waiting time, provider gender, and provision of additional services. Considering that the world is evolving and newer products like Lenacapavir are being introduced and will primarily rely on the established PrEP programs. I have a few follow-up questions and clarifications to the author

*Response: We thank the reviewers for this encouraging overall comment regarding our work.*

2. The study does not specify prior use, but the population likely included both PrEP-experienced and PrEP-naïve individuals. This could affect how preferences were formed. Can the author clarify if this information is available?

Response*: We appreciate the reviewers’ observation. Our study collected data only on current PrEP use, as detailed in Table 3 (condom use, syphilis/HIV testing behaviors, and PrEP use), with 39.4% of participants reporting current oral PrEP use. Although we did not assess prior PrEP use, we acknowledge it may influence formulation preferences. Despite this limitation, we found high willingness to use PrEP among non-current users, with injectable PrEP preferred by 77.3%. Willingness to use was also significantly higher among MSWs compared to FSWs. The following text has been included in the abstract (page 3, lines 13-20), and results (page 11, lines 22)*

**PrEP awareness was high but lower among MSWs (86.5% vs. 95.5%, p = 0.027). Overall, 39.4% reported current PrEP use. Injectable PrEP was the most preferred (77.3%), and willingness to use PrEP among non-users was higher in MSWs (67.3% vs. 45.7%, p = 0.001).**

3. The study was conducted in urban settings; there was no representation from rural areas, which may limit the generalizability to rural sex worker populations and bearing in mind that most PrEP services are in urban settings. This could be highlighted as a study limitation.

*Response: We agree with the reviewer. We explicitly acknowledged this on page 15, lines 9–12, noting that our sample from two cities may not represent all Ugandan MSWs and FSWs, especially those in rural areas.*

4. While participants varied in age and duration in sex work, the study did not explicitly report on gender identity diversity, yet it is a critical point mentioned in this study for determining preferences (e.g., transgender SWs) or levels of mobility and HIV risk, which would be important to determine preferences.

*Response: We value the reviewers’ comment that gender diversity may influence preferences. Our primary objective was to assess CAB-LA delivery preferences among heterosexual male and female sex workers. While we did not explore how mobility or risk levels impact preferences, our findings show that delivery location and additional services were key attributes for both groups. We now present demographics, PrEP awareness, and usage by sex (Tables 2 and 3), and note in the results that both MSWs and FSWs preferred injectable PrEP (page 11, line 12).*

5. While the study did not deeply explore the influence of stigma, harassment, or gendered violence on preferences, these remain critical contextual factors for both access and uptake of services and should be integrated in future research. The preference of services is also determined by access/availability, which also determines uptake

*Response: We appreciate the reviewer's observation and recommendation. This was a limitation of our study. We acknowledge the importance of examining the impact of stigma, harassment, and gender-based violence on preferences in future studies. We have now included this as a limitation, page 15-lines 17-18*

**Furthermore, we did not assess the role of stigma, harassment, or gender-based violence—contextual factors that could influence PrEP preferences.**

5.While private clinics and pharmacies that offer additional health services were the most preferred delivery models for CAB-LA PrEP among both groups. Uganda prides in having a very successful integrated service delivery model called MARPI, which are KP services integrated in the health sector. Did the authors *include such in their questionnaire? This is becoming increasingly important in the current funding landscape, where KP services remain under-prioritized amid a balance between lifesaving and prevention narratives.

*Response: We concur with the reviewers that MARPI, a center of excellence for KP services, is well-known in Uganda.* *While we anticipated that male and female sex workers would prefer this model for CAB-LA PrEP delivery, our analysis (Table 6) indicated that MARPI received a lower ranking (utility score 2.55) than private clinics (utility score 43.3) or private pharmacies (utility score 39.8), as detailed on pages 33-36*

**An integrated service delivery model known as MARPI—a center of excellence for key population services—is widely recognized in Uganda [61].** **Although we anticipated that both male and female sex workers would prefer this model for CAB-LA PrEP delivery, our analysis revealed that MARPI was ranked lower (utility score: 2.55) compared to private clinics (utility score: 43.3) and private pharmacies (utility score: 39.8). This suggests a possible preference for more discreet service delivery settings, where individuals may feel less exposed or less likely to be identified as sex workers [62].**

6. The author highlights the private sector and others as Feasible Options for delivering CAB LA and leveraging private clinics and pharmacies aligns with the study's findings and may be feasible if proper training and regulation are ensured. It would be great for the author to highlight if similar arrangements are available in the country to roll out other public health interventions through the private sector. E.g. Self -testing, private condom dispensing, family planning or even malaria treatment and other services to strengthen the findings.

*Response: We appreciate the reviewers' suggestion to highlight other arrangements for expanding access to other health interventions, such as malaria treatment, condom/contraceptive distribution, and self-testing through private pharmacies in Uganda. In the discussion on page 15, lines 2-3, we note that private pharmacies are a common first point of care and are widely available in Uganda. The following text has been included in the revised manuscript.*

**In Uganda, private pharmacies play a crucial role in distributing products for preventing malaria, tuberculosis (TB), and HIV. They provide artemisinin-based combination therapies, malaria rapid diagnostic kits, HIV self-test kits, pre-exposure prophylaxis, condoms, and emergency contraceptives.** **However, pharmacy-based delivery models may present cost barriers, particularly when SWs prefer integrated services**

7. The study used trained interviewers and implemented informed consent protocols to ensure ethical participation. However, the methods section does not detail protections from harm, and ethical safeguards which were likely approved by local ethics boards given the vulnerable population involved.

*Response: We apologize for not providing details about the safeguards. The REC indeed approved these measures, including prior ethical training, guidelines for maintaining our researcher role, community entry protocols, interview venue selection, and respondent safety procedures. We have now updated the text to include these safeguards. The text below has been included (page 9, lines 12-17);*

**To ensure respondent safety, our research team completed ethics training before recruitment, engaged with sex worker leaders, established safety protocols (such as working in pairs on the street and sharing contact information), sought participant community input on safety, and conducted procedures at respondent-chosen venues. Data was anonymized, with no identifiers or names used, to maintain confidentiality and anonymity.**

8.I find it surprising for the findings that give minimal importance to provider gender and wait time challenges which are common assumptions and underscores that accessibility and comprehensiveness matter more to this. population. Could the author confirm this?

*Response: We agree with the reviewers that provider gender and clinic waiting time may influence preferences. However, our study revealed that dispensing location (80% relative importance) and additional services (12%) were the most valued attributes for CAB-LA delivery, while provider gender/cadre (4.8%) and waiting time (3.2%) were considered least important (Table 4).* *Nevertheless, the three top-ranked CAB-LA delivery models prioritized access through private pharmacies or clinics with short waiting times, female or peer providers, and integrated health services (abstract page 4, lines 1-4).*

*Also, in the discussion page 14*

**Additionally, the top three preferred CAB-LA delivery models emphasized access through private facilities, with short waiting times, female or peer providers, and integrated health services. The most preferred model was a private clinic with a waiting time under one hour, attended by a female peer provider, and offering psychosocial support. This was followed by a private pharmacy providing cancer screening, staffed by a female health worker, also with a waiting time under one hour. The third-ranked model involved a private pharmacy with a female peer provider, provision of contraceptives or condoms, and a waiting time under two hours. Ranked fourth was a family planning clinic offering screening for diabetes and hypertension, attended by a female provider, with a waiting time under two hours.**

*Then page 14 (lines 11-13)*

**The preference for CAB-LA delivery through private facilities, with short waiting times, female or peer providers, and integrated services highlights key priorities for effective rollout among SWs**

Reviewer #2:

1. This study aimed to understand the preferences for long-acting injectable cabotegravir (CAB-LA) among HIV-negative male and female sex workers (MSWs and FSWs) in Uganda using a discrete choice experiment (DCE). The findings showed MSWs and FSWs both prioritized dispensing location and provision of additional services. This study has some merits, but several concerns needed to be addressed before publication.

Response: We thank the reviewer for this encouraging comment regarding our study.

2. Abstract: The Results section does not seem to present all the important findings of the study, including the preferred delivery model. In addition, the final sentence appears to be a repetition of the previous one; please make it more concise. Based on the current results, it is difficult to conclude that 'introducing injectable PrEP in Uganda could boost acceptability and uptake among sex workers'. Please clarify this.

*Response: We agree with the reviewer that important findings were not included in the abstract due to word limit concerns. We apologize for the unclear conclusion. We have now added details on PrEP use and preferences, and revised the conclusion for better clarity. The following text has been added (page 3, lines 17-20).*

**PrEP awareness was high but lower among MSWs (86.5% vs. 95.5%, p = 0.027). Overall, 39.4% reported current PrEP use. Injectable PrEP was most preferred (77.3%), and willingness to use PrEP among non-users was higher in MSWs (67.3% vs. 45.7%, p = 0.001)**

*We have also included the following text in the Abstract regarding the model (page 4, lines 1-2).*

**The three top-ranked CAB-LA delivery models emphasized access through private pharmacies or clinics with short waiting times, female or peer providers, and integrated health services.**

*Additionally, we have revised the conclusion, and it now reads as follows (page 4, lines 6-9):*

**Expanding injectable PrEP through private sector channels may improve access among SWs. Still, implementation research is needed to guide integration of supportive services and SWs’ willingness to pay or co-pay for pharmacy or private clinic-based delivery.**

2. Methods: The literature review revealed several key attributes influencing decision-making regarding HIV prevention service uptake. However, the authors finally only included four of them without any clear explanations. Please clarify this. In addition, the authors mentioned 16 hypothetical choice sets, but you didn’t clarify how to get these 16 choices sets. Please provide the formula of the rule of thumb and clarify how to get the sample size.

*Response: We thank the reviewers for this insightful observation and apologize for not providing a clear rationale for our attribute selection. While we initially considered a broad range of service characteristics—including service location, waiting time, provider gender, provider attitude, availability of medical supplies, distance, cost, and the provision of additional services—we ultimately focused on four attributes: location, provider gender, waiting time, and the provision of additional services. This selection was informed by prior studies that identified these as key factors influencing preferences for service delivery.*

**However, as CAB-LA had not yet been rolled out, we hypothesized that delivery preferences would be shaped by service characteristics such as location, waiting time, provider gender, and availability of additional services.**

*Secondly, we reported that prior studies had shown that 16 choice sets could be overly burdensome for respondents. We apologize if this was unclear; in our study, we used 10 choice sets, not 16. This decision was made to reduce respondent burden and minimize mental fatigue during the discrete choice experiment. The following text has been included; (page 7 lines 18-22);*

**To minimize respondent fatigue, we aimed to include fewer choice sets. Using STATA version 17.0, we developed a statistically efficient and balanced fractional factorial DCE design comprising 10 choice cards. Each card included four alternative service delivery options and an opt-out option, based on four key service attributes: location, waiting time, provider gender, and provision of additional services,**

*Regarding sample size, we have now included Johnson and Orme’s rule of thumb formula that we used to estimate the sample size on page 8, line 13.*

**N ≥500 Lmx/JS**

**where Lmx is the maximum number of levels across all attributes, J is the number of alternatives per choice set (excluding the opt-out), and S is the number of choices sets per respondent. In this study, the attribute location had the most levels (Lmax = 8), with 4 alternatives per choice set (J = 4) and 10 choice sets per respondent (S = 10). Applying the formula yielded a minimum sample size of 100 respondents. To accommodate an anticipated 20% non-response or opt-out rate, we aimed to enroll 125 participants per site. We enrolled 124 and 127 respondents, respectively, from Mbarara and Kampala, using snowball sampling from previously identified sex work hotspots and an established cohort of MSWs and FSWs.**

3. Discussion: The authors only discussed the most preferred attribute, failing to address other findings, such as the preferred delivery model. In addition, the results should be compared to evidence from other parts of the world, not just SSA.

*Response: We have revised the discussion to include the preferred delivery model, as discussed on pages 12-15. The revised discussion now reads as follows*:

**Discussion**

**In this DCE exploring CAB-LA PrEP delivery preferences among MSWs and FSWs in Uganda, most respondents (77.3%) preferred CAB-LA over other PrEP options. Among non- current PrEP users, willingness to initiate PrEP was higher among MSWs (67.3%) than FSWs (45.7%). However, both FSWs and MSWs prioritized receiving CAB-LA from private clinics or pharmacies that provide additional services such as psychosocial support, risk reduction counseling, and cancer screening. Additionally, the top three preferred CAB-LA delivery models emphasized access through private facilities with short waiting times, female or peer providers, and integrated health services. The most preferred model was a private clinic with a waiting time less than one hour, attended by a female peer provider, and offering psychosocial support. This was followed by a private pharmacy providing cancer screening, staffed by a female health worker, also with a waiting time of under one hour. The third-ranked model involved a private pharmacy with a female peer provider, provision of contraceptives or condoms, and a waiting time under two hours. Ranked fourth was a family planning clinic offering screening for diabetes and hypertension, attended by a female provider, with a waiting time under two hours.**

**The strong preference for CAB-LA observed in this cohort suggests that the introducing injectable PrEP options could increase PrEP acceptability and uptake among SWs in Uganda. Expanding the variety of PrEP modalities has been shown to improve user choice, acceptability, and adherence [43-45]. Emerging options such as Injectable lenacapavir, administered only twice a year [46, 47] might further increase acceptability among SWs, by offering even a more convenient and less frequent dosing schedule than CAB-LA PrEP. Although limited data exist on PrEP preferences among MSWs, similar preferences for injectable PrEP options have been reported globally among FSWs, MSM, and other key populations [48-54]. Studies conducted in SSA—specifically in Kenya, Tanzania, South Africa, and Malawi—among FSWs and vulnerable youth report high preference rates (74–88%) for injectable PrEP [21, 55-57]. While we did not explore reasons for this preference, prior studies suggest that ease, convenience, perceived effectiveness, and privacy make injectable PrEP appealing to SWs [53, 58, 59].**

**Our finding that willingness to initiate PrEP was higher among MSWs than FSWs may reflect greater perceived HIV risk. In this study, MSWs reported lower rates of condom use and consistency compared to FSWs, consistent with our previous findings [3].**

**Consistent with prior SSA studies on oral PrEP, dispensing location, and additional services were highly valued by FSWs and adolescent girls [60-62]. In our study, both MSWs and FSWs preferred to access CAB-LA PrEP from private pharmacies and private clinics offering additional services, highlighting private sector channels as an alternative strategy for scaling up injectable PrEP services. The preference for CAB-LA delivery through private facilities, with short waiting times, female or peer providers, and integrated services highlights key priorities for effective rollout among SWs [31, 63]. Engaging private pharmacies, ensuring client-centered and stigma-free care, and offering integrated health services [64, 65] may improve acceptability and uptake. An integrated service delivery model known as MARPI—a center of excellence for key population services—is widely recognized in Uganda [66]. Although we anticipated that both male and female sex workers would prefer this model for CAB-LA PrEP delivery, our analysis revealed that MARPI was ranked lower (utility score: 2.55) compared to private clinics (utility score: 43.3) and private pharmacies (utility score: 39.8). This suggests a possible preference for more discreet service delivery settings, where individuals may feel less exposed or less likely to be identified as sex workers [67]. Notably, the private pharmacy model has already been scaled up successfully in Kampala for ART delivery [68], consistent with prior evidence on the acceptability of private pharmacies for HIV prevention services in SSA [29]. Community pharmacies in the US have successfully implemented PrEP programs, with a notable example being a Seattle study where CAB-LA PrEP delivery achieved 94% on-time injection adherence and 81% retention in care at 26 weeks [69, 70]. Our study however, highlights the importance of integrating additional services, such as psychosocial support, risk reduction counseling, and cancer screening, into pharmacy-based CAB-LA PrEP delivery models, similar to Malawi, where FSWs expressed a desire for oral PrEP services bundled with cervical cancer screening at NGO-run clinics [21]. In Uganda, private pharmacies play a crucial role in distributing products for preventing malaria, tuberculosis (TB), and HIV. They provide artemisinin-based combination therapies, malaria rapid diagnostic kits, HIV self-test kits, pre-exposure prophylaxis, condoms, and emergency contraceptives and ART refills [68, 71-75] . However, pharmacy-based delivery models may present cost barriers, particularly when SWs prefer integrated services.**

**Implementation research and costing studies are needed to evaluate the feasibility, affordability, and sustainability of such models. Additionally, public sector services remain a key platform for HIV prevention and may require adaptation to improve efficiency, provider attitudes, and integration of services [64, 65] to better meet the needs of SWs.**

**The present study's strengths include being the first to investigate preferences for CAB-LA PrEP delivery among MSWs and FSWs in Uganda and using a DCE to provide insights/delivery strategies that can inform future implementation. However, limitations exist: the sample from two cities may not represent all Ugandan MSWs and FSWs, especially those living in more rural settings. Results may also not apply to other African settings; in Malawi, FSWs preferred family planning clinics or NGO-run drop-in centers for oral PrEP [60]. DCEs' hypothetical scenarios may not mirror real-world choices. Additionally, we did not explore the reasons why SWs in this cohort preferred private pharmacies and clinics. Furthermore, we did not assess the role of stigma, harassment, or gender-based violence—contextual factors that could influence PrEP preferences. Despite these limitations, our findings align with studies on oral PrEP delivery preferences [21, 76].**

**Conclusions**

**In this cohort, willingness to initiate PrEP was high among MSWs. Expanding injectable PrEP through private sector channels may improve access among sex workers. However, implementation research is needed to guide integration of supportive services and assess willingness to pay for pharmacy- or clinic-based delivery.**

4. Conclusions: It is difficult to conclude the first sentence base on the current results or the discussion.

*Response: We appreciate the reviewer's observation. We have now revised the text on page 15, line 20:*

**In this cohort, willingness to initiate PrEP was high among MSWs. Expanding injectable PrEP through private sector channels could improve access among sex workers. However, implementation research is needed to guide integration of supportive services and assess willingness to pay for pharmacy- or clinic-based delivery.**

Reviewer #3:

1. Thank you for the opportunity to review the submission PGPH-D-25-01202 entitled “Preferences for Long-Acting Injectable HIV Pre-exposure Prophylaxis Service Delivery Among Male and Female Sex Workers in Uganda: A Discrete Choice Experiment.” This paper analyzes a sample of 251 sex workers regarding long-acting injectable cabotegravir (CAB-LA) using a discrete choice experiment (DCE). Overall, I think this is important work that contributes significantly to the existing literature. I do have some suggestions for revisions that I think would enhance impact of the manuscript.

*Response: We thank the reviewers for this uplifting comment that shares our view that the findings of this study are important to PrEP rollout.*


2. Background: Authors establish that sex workers are an important group in HIV prevention efforts and that CAB-LA has the potential for preventing HIV in this population. They highlight the lack of data on male sex workers (MSWs). While data exists for preferences for daily oral PrEP delivery, there is less regarding CAB-LA. This sufficiently establishes the significance of the current study

*Response: We thank the reviewers for this encouraging comment regarding our background*

3. Method: I think the discussion of underlying theories of choice may be moved into the background.

*Response:* *We thank the reviewers for this valuable suggestion and have implemented it as recommended in the revised manuscript on pages 5, lines 15-23 and page 6, lines 1-12.*

4.The team’s previous literature review regarding service preferences may also be useful information in the background on page 6, lines 13-21

*Response: We thank the reviewers for this valuable suggestion and have implemented it as recommended in the revised manuscript*

Results:

4. Differences are largely discussed between sites in the bivariates. Given the emphasis on MSWs, I wonder if it would be useful to report any significant differences in bivariate associations between MSWs and FSWs.

*Response: We thank the reviewer for this suggestion. We have implemented it in the Results (Table 2 and Table 3)*

Table 2: Characteristics of SWs enrolled in the DCE survey stratified by sex

| **Characteristic** | **Overall, N (Col%)** | **Sex** | | **p-value** |
| --- | --- | --- | --- | --- |
|  |  | **Female, N (Col%)** | **Male, N (Col%)** |  |
| ***Overall*** | *251* | *199 (79.3%)* | *52 (20.7%)* |  |
| **Location** |  |  |  | 0.683^C^ |
| **Kampala** | 127 (50.6) | 102 (51.3) | 25 (48.1) |  |
| **Mbarara** | 124 (49.4) | 97 (48.7) | 27 (51.9) |  |
| **Age in completed years** | 25 (22, 31) | 25 (22, 32) | 25 (22, 29) | 0.367^M^ |
| **Highest Level of Education Attained** |  |  |  | <0.001^F^ |
| **Higher education** | 23 (9.2) | 9 (4.5) | 14 (26.9) |  |
| **No education** | 11 (4.4) | 9 (4.5) | 2 (3.8) |  |
| **Primary** | 107 (42.6) | 92 (46.2) | 15 (28.8) |  |
| **Secondary** | 110 (43.8) | 89 (44.7) | 21 (40.4) |  |
| **Marital Status** |  |  |  | 0.360^F^ |
| **Married** | 18 (7.2) | 12 (6.0) | 6 (11.5) |  |
| **Regular boyfriend/girlfriend** | 45 (17.9) | 34 (17.1) | 11 (21.2) |  |
| **Single** | 183 (72.9) | 149 (74.9) | 34 (65.4) |  |
| **Widow** | 5 (2.0) | 4 (2.0) | 1 (1.9) |  |
| **Number of Children (biological) Currently** | 1 (0, 2) | 1 (0, 2) | 1 (0, 1) | <0.001^M^ |
| **Duration in sex work (In completed months)** | 24 (12, 48) | 24 (12, 48) | 18 (5, 36) | 0.009^M^ |
| **Description of current work** |  |  |  | <0.001^F^ |
| **Full-time, to supplement my other sources of income** | 8 (3.2) | 5 (2.5) | 3 (5.8) |  |
| **Full time, as I have no other sources of income** | 159 (63.3) | 143 (71.9) | 16 (30.8) |  |
| **Part-time, as I am a student** | 4 (1.6) | 1 (0.5) | 3 (5.8) |  |
| **Part-time, as I have other sources of income** | 80 (31.9) | 50 (25.1) | 30 (57.7) |  |
| **Average number of clients per week** | 23 (9, 34) | 26 (20, 35) | 3 (3, 5) | <0.001^M^ |
| **Average amount of money earned per week (In Ugx)** | 200,000 (145,000, 350,000) | 250,000 (155,000, 400,000) | 145,000 (60,000, 250,000) | <0.001^M^ |

Table 3: Condom use, syphilis, HIV testing behaviors, and PrEP use stratified by sex

| **Characteristic** | **Overall, N (Col%)** | **sex** | | **P-value** |
| --- | --- | --- | --- | --- |
|  |  | **Female, N (Col %)** | **Male, N (Col %)** |  |
| ***Overall*** | ***251*** | ***199 (79.3)*** | ***52 (20.7)*** |  |
| **Used condom at last sex** |  |  |  | <0.001^C^ |
| **No** | 32 (12.7) | 15 (7.5) | 17 (32.7) |  |
| **Yes** | 219 (87.3) | 184 (92.5) | 35 (67.3) |  |
| **Description of condom use** |  |  |  | <0.001^F^ |
| **Consistently use at all times with all clients** | 138 (55.0) | 121 (60.8) | 17 (32.7) |  |
| **Sometimes non-use for more money** | 27 (10.8) | 23 (11.6) | 4 (7.7) |  |
| **Sometimes non-use if I like the client by look** | 7 (2.8) | 5 (2.5) | 2 (3.8) |  |
| **Sometimes non-use if the client requests so** | 9 (3.6) | 7 (3.5) | 2 (3.8) |  |
| **Sometimes non-use if we test for HIV** | 30 (12.0) | 20 (10.1) | 10 (19.2) |  |
| **Sometimes non-use with regular clients** | 39 (15.5) | 23 (11.6) | 16 (30.8) |  |
| **Other¶** | 1 (0.4) | 0 (0) | 1 (1.9) |  |
| **Syphilis testing frequency (prior 6 months) (median [IQR])** | 1 (0, 2) | 1 (0, 2) | 1 (1, 2) | 0.018^M^ |
| **HIV testing frequency (prior months) (median [IQR])** | 2 (1, 3) | 2 (1, 3) | 2 (2, 3) | 0.18^M^ |
| **HIV testing modality (prior 6 months)** |  |  |  | 0.59^F^ |
| **Not tested in prior 6 months** | 5 (2.0) | 5 (2.5) | 0 (0.0) |  |
| **Outreach** | 77 (30.7) | 60 (30.2) | 17 (32.7) |  |
| **Self-testing** | 51 (20.3) | 38 (19.1) | 13 (25.0) |  |
| **Visited a health facility** | 118 (47.0) | 96 (48.2) | 22 (42.3) |  |
| **Intention to test for HIV in the next 3 months** |  |  |  | 0.006^F^ |
| **High intention** | 181 (72.1) | 152 (76.4) | 109 (87.9) |  |
| **Low intention** | 43 (17.1) | 29 (14.6) | 9 (7.3) |  |
| **No intention** | 18 (7.2) | 14 (7.0) | 1 (0.8) |  |
| **Not sure** | 9 (3.6) | 4 (2.0) | 5 (4.0) |  |
| **Intention to take a serological syphilis test in the next 3 months** |  |  |  | 0.669^F^ |
| **High intention** | 136 (54.2) | 107 (53.8) | 29 (55.8) |  |
| **Low intention** | 63 (25.1) | 48 (24.1) | 15 (28.8) |  |
| **No intention** | 42 (16.7) | 36 (18.1) | 6 (11.5) |  |
| **Not sure** | 10 (4.0) | 8 (4.0) | 2 (3.8) |  |
| **Awareness of HIV PrEP** |  |  |  | 0.027^F^ |
| **Yes** | 235 (93.6) | 190 (95.5) | 45 (86.5) |  |
| **Prior CAB LA awareness (N = 235)** |  |  |  | 0.457^C^ |
| **Yes** | 17 (7.2) | 12 (6.3) | 5 (11.1) |  |
| **Currently using PrEP use (N = 235)** |  |  |  | <0.54^C^ |
| **Yes, oral** | 99 (39.4) | 80 (42.1) | 19 (42.2) |  |
|  |  |  |  | 0.614^F^ |
| **Preferred PrEP option** |  |  |  |  |
| **Daily oral** | 44 (17.5) | 34 (17.1) | 10 (19.2) |  |
| **Injectable** | 194 (77.3) | 156 (78.4) | 38 (73.1) |  |
| **Vaginal ring** | 4 (1.6) | 3 (1.5) | 1 (1.9) |  |
| **On-demand** | 9 (3.6) | 6 (3.0) | 3 (5.8) |  |
| **Willingness to use PrEP (none-current user), N = 152** |  |  |  | 0.001^C^ |
| **Very high or high** | 126 (82.9%) | 54 (45.7) | 22 (67.3%) |  |

5. I am assuming it’s not possible to examine the differences between MSW and FSW preferences in the model. (I am not as familiar with DCE). If it is possible, I would suggest commenting on this as it is sold as a strength of the study.

*Response: We thank the reviewer for this suggestion. In our analysis under Table 5: Results of random parameter logit model (mixed logit model) for the CAB LA PrEP model attributes* by sex, we show that both MSWs and FSW attached more importance to location and additional services. We were unable do the sub-analysis for the model, but we have included the results and discussion that the three top-ranked CAB-LA delivery models emphasized access through private pharmacy or clinic, with short waiting times, female or peer providers, and integrated health services.*

|  | **MALES** | | | **FEMALES** | | |
| --- | --- | --- | --- | --- | --- | --- |
| **Attributes and levels^a^** | **Coefficients** | **SD** | **Relative importance¶** | **Coefficients** | **SD** | **Relative importance¶** |
| Constant | -1.33 |  |  | -1.26 | - |  |
| **Dispensing location** |  |  | **81.1%** |  |  | **82.9%** |
| *Private drug shops* | *Reference* |  |  | *Reference* |  |  |
| ART clinic | 7.05 | <0.01 |  | 6.77 | 0.11 |  |
| Private clinic | 31.72 | <0.01 |  | 40.69 | <0.01 |  |
| NGO-run drop-in center | 8.20 | <0.01 |  | 7.79 | 0.03 |  |
| Private pharmacy | 29.40 | <0.01 |  | 38.29 | 0.01 |  |
| Family planning clinic | 7.91 | 0.01 |  | 7.78 | 0.13 |  |
| District hospital | 4.67 | <0.01 |  | 4.37 | 0.11 |  |
| STI clinic | 5.23 | 0.01 |  | 5.33 | 0.53 |  |
| Community health center | 5.14 | 0.01 |  | 4.90 | 0.34 |  |
| MARPs clinic | 6.95 | 0.01 |  | 7.44 | 1.28 |  |
| NGO-run mobile outreach | 5.17 | <0.01 |  | 4.87 | 0.25 |  |
| **Clinic waiting time** |  |  | **3.9%** |  |  | **4.0%** |
| < 1 hour | 1.04 | <0.01 |  | 1.42 | 0.26 |  |
| 1 hour | -0.71 | 0.03 |  | -0.56 | 0.07 |  |
| *2 hours* | *Reference* |  |  | *Reference* | *Reference* |  |
| **Provider** |  |  | **4.2%** |  |  | **4.0%** |
| Male, HCW | Reference |  |  | *Reference* | *Reference* |  |
| Female, HCW | -0.96 | 0.01 |  | -0.85 | 0.27 |  |
| Male, peer | -0.16 | 0.02 |  | -0.02 | 0.04 |  |
| Female, peer | -1.88 | 0.03 |  | -1.97 | 0.15 |  |
| **Additional services at the site** |  |  | **10.8%** |  |  | **9.0%** |
| *Receiving contraceptive services e.g. condoms* | *Reference* |  |  | *Reference* | *Reference* |  |
| Psychosocial support | 4.83 | <0.01 |  | 4.85 | 0.25 |  |
| Check for hypertension and diabetes | 3.79 | 0.05 |  | 4.03 | 0.67 |  |
| Check for cancer i.e. cervical or prostate | 4.26 | <0.01 |  | 4.44 | 0.75 |  |
| Receive risk counseling | 1.15 | 0.03 |  | 1.29 | 0.43 |  |

Discussion:

6. I would suggest discussing MSW a bit more in the discussion, as this is a novel aspect of the study that is emphasized in the background.

*Response: We sincerely thank the reviewer for this suggestion. We have implemented it in the discussion section. Our discussion (pages 13-15) has now been revised to read as follows*;

**Discussion**

**In this DCE exploring CAB-LA PrEP delivery preferences among MSWs and FSWs in Uganda, most respondents (77.3%) preferred CAB-LA over other PrEP options. Among non- current PrEP users, willingness to initiate PrEP was higher among MSWs (67.3%) than FSWs (45.7%). However, both FSWs and MSWs prioritized receiving CAB-LA from private clinics or pharmacies that provide additional services such as psychosocial support, risk reduction counseling, and cancer screening. Additionally, the top three preferred CAB-LA delivery models emphasized access through private facilities with short waiting times, female or peer providers, and integrated health services. The most preferred model was a private clinic with a waiting time less than one hour, attended by a female peer provider, and offering psychosocial support. This was followed by a private pharmacy providing cancer screening, staffed by a female health worker, also with a waiting time of under one hour. The third-ranked model involved a private pharmacy with a female peer provider, provision of contraceptives or condoms, and a waiting time under two hours. Ranked fourth was a family planning clinic offering screening for diabetes and hypertension, attended by a female provider, with a waiting time under two hours.**

**The strong preference for CAB-LA observed in this cohort suggests that the introducing injectable PrEP options could increase PrEP acceptability and uptake among SWs in Uganda. Expanding the variety of PrEP modalities has been shown to improve user choice, acceptability, and adherence [43-45]. Emerging options such as Injectable lenacapavir, administered only twice a year [46, 47] might further increase acceptability among SWs, by offering even a more convenient and less frequent dosing schedule than CAB-LA PrEP. Although limited data exist on PrEP preferences among MSWs, similar preferences for injectable PrEP options have been reported globally among FSWs, MSM, and other key populations [48-54]. Studies conducted in SSA—specifically in Kenya, Tanzania, South Africa, and Malawi—among FSWs and vulnerable youth report high preference rates (74–88%) for injectable PrEP [21, 55-57]. While we did not explore reasons for this preference, prior studies suggest that ease, convenience, perceived effectiveness, and privacy make injectable PrEP appealing to SWs [53, 58, 59].**

**Our finding that willingness to initiate PrEP was higher among MSWs than FSWs may reflect greater perceived HIV risk. In this study, MSWs reported lower rates of condom use and consistency compared to FSWs, consistent with our previous findings [3].**

**Consistent with prior SSA studies on oral PrEP, dispensing location, and additional services were highly valued by FSWs and adolescent girls [60-62]. In our study, both MSWs and FSWs preferred to access CAB-LA PrEP from private pharmacies and private clinics offering additional services, highlighting private sector channels as an alternative strategy for scaling up injectable PrEP services. The preference for CAB-LA delivery through private facilities, with short waiting times, female or peer providers, and integrated services highlights key priorities for effective rollout among SWs [31, 63]. Engaging private pharmacies, ensuring client-centered and stigma-free care, and offering integrated health services [64, 65] may improve acceptability and uptake. An integrated service delivery model known as MARPI—a center of excellence for key population services—is widely recognized in Uganda [66]. Although we anticipated that both male and female sex workers would prefer this model for CAB-LA PrEP delivery, our analysis revealed that MARPI was ranked lower (utility score: 2.55) compared to private clinics (utility score: 43.3) and private pharmacies (utility score: 39.8). This suggests a possible preference for more discreet service delivery settings, where individuals may feel less exposed or less likely to be identified as sex workers [67]. Notably, the private pharmacy model has already been scaled up successfully in Kampala for ART delivery [68], consistent with prior evidence on the acceptability of private pharmacies for HIV prevention services in SSA [29]. Community pharmacies in the US have successfully implemented PrEP programs, with a notable example being a Seattle study where CAB-LA PrEP delivery achieved 94% on-time injection adherence and 81% retention in care at 26 weeks [69, 70]. Our study however, highlights the importance of integrating additional services, such as psychosocial support, risk reduction counseling, and cancer screening, into pharmacy-based CAB-LA PrEP delivery models, similar to Malawi, where FSWs expressed a desire for oral PrEP services bundled with cervical cancer screening at NGO-run clinics [21]. In Uganda, private pharmacies play a crucial role in distributing products for preventing malaria, tuberculosis (TB), and HIV. They provide artemisinin-based combination therapies, malaria rapid diagnostic kits, HIV self-test kits, pre-exposure prophylaxis, condoms, and emergency contraceptives and ART refills [68, 71-75]. However, pharmacy-based delivery models may present cost barriers, particularly when SWs prefer integrated services. Implementation research and costing studies are needed to evaluate the feasibility, affordability, and sustainability of such models. Additionally, public sector services remain a key platform for HIV prevention and may require adaptation to improve efficiency, provider attitudes, and integration of services [64, 65] to better meet the needs of SWs.**

**The present study's strengths include being the first to investigate preferences for CAB-LA PrEP delivery among MSWs and FSWs in Uganda and using a DCE to provide insights/delivery strategies that can inform future implementation. However, limitations exist: the sample from two cities may not represent all Ugandan MSWs and FSWs, especially those living in more rural settings. Results may also not apply to other African settings; in Malawi, FSWs preferred family planning clinics or NGO-run drop-in centers for oral PrEP [60]. DCEs' hypothetical scenarios may not mirror real-world choices. Additionally, we did not explore the reasons why SWs in this cohort preferred private pharmacies and clinics. Furthermore, we did not assess the role of stigma, harassment, or gender-based violence—contextual factors that could influence PrEP preferences. Despite these limitations, our findings align with studies on oral PrEP delivery preferences [21, 76].**

**Conclusions**

**In this cohort, willingness to initiate PrEP was high among MSWs. Expanding injectable PrEP through private sector channels could improve access among sex workers. However, implementation research is needed to guide integration of supportive services and assess willingness to pay for pharmacy- or clinic-based delivery**

7. It may be worth discussing the implications of a private vs. public pharmacy in the context of Uganda. This is the preference, but does it have implications like a higher fee? Are private pharmacies as available as public pharmacies (e.g. equivalently distributed)?

*Response:*

*Thank you for this observation. We have noted in the discussion that, In Uganda, private pharmacies play a crucial role in distributing products for preventing malaria, tuberculosis (TB), and HIV. They provide artemisinin-based combination therapies, malaria rapid diagnostic kits, HIV self-test kits, pre-exposure prophylaxis, condoms, and emergency contraceptives and ART refills. However, we are unsure what is meant by "public pharmacy," as Uganda primarily has hospital-based pharmacies within public health facilities. We are uncertain whether the reviewer was referring to these hospital pharmacies. Nonetheless, we have indicated that private pharmacy-based delivery models may present cost-related barriers, particularly when integrated services are preferred by sex workers. We have also highlighted the need for further research on integration and cost considerations within this delivery model.*

8. The findings point to integrated clinical settings and the authors point to a similar model in Malawi. Do such clinics exist in Uganda? I think more comment on the current feasibility or infrastructure in Uganda would be helpful context for this finding.

*Response: We thank the reviewer for this observation. Indeed, integrated models are limited in our context despite research showing their preference by the SWs. It is on this basis that we recommend further implementation studies to look into the feasibility of such integrative models in private pharmacies.*

Overall:

9. I think this is an important study that contributes important information to the field.

*Response: We thank the reviewer for this insightful comment regarding our work.*

10. I would recommend emphasizing the MSWs in the results and discussion more, as this is a novel component of the study that is emphasized in the background section. As currently presented I feel a bit “let down” as a reader, because I was expecting more commentary on the MSWs based on the background section as presented.

*Response: We are grateful to the reviewer for this suggestion. We have tried to comment throughout the results and discussion on MSWs vs. FSWs.*

We thank the reviewers for their helpful comments, which have improved the quality of our manuscript. We trust that the revised manuscript will be suitable for acceptance, but we are happy to consider further revisions if necessary.

Sincerely

Richard Muhindo, PhD, MPH, BSN

**Senior Lecturer**, Department of Nursing, College of Health Sciences

Makerere University

Corresponding author

Email: r.muhindo@yahoo.com
