# Supplementary material for: Preferences for long-acting injectable HIV pre-exposure prophylaxis service delivery among male and female sex workers in Uganda: A discrete choice experiment
Source: PLOS Glob Public Health. 2025 Oct 9;5(10):e0004798. doi: 10.1371/journal.pgph.0004798 (PMC12510523; doi:10.1371/journal.pgph.0004798)
Supplement: Attachment — Submitted filename: Response to Reviewers.doc [file pgph.0004798.s006.doc]

**Makerere	University**

P.O. Box 7072 Kampala, Uganda	**Tel:** 256 414 530404

**E-mail**: [nursing@chs.mak.ac.ug](mailto:nursing@chs.mak.ac.ug)

**SCHOOL OF HEALTH SCIENCES**

**Department of Nursing**

September 1st, 2025

Dr. Johanna Pruller

Staff Editor, Plos Global Public Health

Dear Dr. Johanna,

Regarding PGPH-D-25-01202R1 - EMID: 9fc7aceccf0b3799

We sincerely thank you for the careful review of our manuscript “*Preferences for long-acting injectable HIV pre-exposure prophylaxis service delivery among male and female sex workers in Uganda: A discrete choice experiment*” and for the constructive feedback provided. We greatly appreciate the encouraging comments and the recognition that our study findings are important to inform the delivery of injectable PrEP among sex workers. We have carefully considered all the comments and revised the manuscript accordingly. Below, we provide a point-by-point response. Reviewer comments are presented in italic, followed by our responses in regular font.

**Reviewer Comments**

Reviewer #1:

1. The paper reads very well and is relevant to the current public health body of knowledge. The study is also timely, novel, and policy-relevant, addressing injectable CAB-LA PrEP among sex workers in Uganda.

*Response: We thank the reviewer for this encouraging overall comment regarding our work.*

2. The introduction could be strengthened by linking the HIV burden among sex workers with the promise of CAB-LA.

Response*: We thank the reviewer for the suggestion to strengthen our introduction. We have tried to link HIV burden and the promise of CAB-LA; the introduction now reads as;*

**Globally, sex workers (SWs) bear a disproportionate burden of HIV, accounting for 7.7% of new infections between 2010 and 2022, despite comprising only a small fraction of the general adult population (1). In Sub-Saharan Africa (SSA), the epicenter of the HIV epidemic, SWs contribute an estimated 5.2%-15% of new infections in 2022 (1).In Uganda, HIV prevalence among female sex workers (FSWs) is particularly high, estimated at 31% (1, 2). In contrast, the epidemiology of HIV among heterosexual male sex workers (MSWs) in SSA, including Uganda, remains poorly understood, with limited data suggesting inconsistent condom use and low HIV testing uptake among MSWs (3).**

**Pre-exposure prophylaxis (PrEP) has been shown in clinical trials to substantially reduce the risk of HIV acquisition, offering SWs an additional effective prevention strategy (4, 5). The World Health Organization (WHO) recommended oral tenofovir disoproxil (TDF)-based PrEP in 2015, and Uganda adopted it as part of its HIV prevention strategy in 2017 (6, 7). Although oral PrEP services are available in Uganda for high-risk individuals, including sex workers (7), PrEP uptake, adherence, and persistence remain suboptimal (8-11). Long-acting formulations that eliminate the need for daily pills offer a promising alternative by providing sustained protection with infrequent dosing and may increase PrEP adoption among FSWs and MSWs. Long-acting injectable cabotegravir PrEP (CAB-LA) has shown superior efficacy in preventing HIV infections, with a 79% reduction in HIV acquisition in new cases observed across over 60 sites, including 20 Sub-Saharan Africa sites, among cisgender women, men who have sex with men, and transgender women who have sex with men (12-15). By reducing the reliance on daily adherence, CAB-LA has the potential to overcome barriers that have limited the impact of oral PrEP and may be particularly well-suited to high-risk populations such as FSWs and MSWs.**

**The WHO endorsement of CAB-LA as a PrEP option (16) and its adoption by Uganda in 2022 (7) have generated optimism for improved HIV prevention outcomes ahead of its rollout. Nevertheless, uncertainty remains regarding the optimal service attributes and delivery models that best facilitate uptake among MSWs and FSWs. Service delivery features significantly influence an individual's decision to utilize a service (17, 18). Economic theories may help explain health consumer choices. First, Lancaster’s Theory of Consumer Choice suggests that consumers derive utility not from the goods or services themselves, but from the attributes or characteristics they offer. [17, 18]. In the context of CAB-LA PrEP, service delivery characteristics may influence uptake decisions among female sex workers (FSWs) and male sex workers (MSWs). According to Lancaster’s Theory of Consumer Choice, individuals derive utility not from the service itself, but from the combination of attributes offered by a particular delivery model [18]. Consequently, individuals choose one service delivery model over another based on the combination of attributes that best aligns with their preferences. To optimize CAB-LA PrEP uptake among MSWs and FSWs, it is essential to identify the delivery attributes that facilitate uptake and to design strategies that reduce barriers while maximizing perceived utility.**

**The second theory, Random Utility Theory (RUT), proposes that an individual’s utility from choosing a particular option consists of two components: a systematic (observable) component, determined by the attributes of the option, and a random (unobservable) component, which captures the influence of unobserved factors such as individual preferences, attitudes, or random error (19, 20). In the context of CAB-LA PrEP, an individual’s decision to select a particular service delivery model is influenced by both observable attributes (e.g., location, provider gender, frequency of visits) and unobservable factors such as personal preferences and attitudes toward healthcare providers.**

**Research in SSA has documented preferred attributes for oral PrEP delivery among FSWs (21), including convenient dispensing locations and access to additional services, such as cervical cancer screening, at family planning clinics and NGO-run drop-in centers (21). In Uganda, FSWs prefer receiving oral PrEP from healthcare workers at clinics, supplemented by short message service (SMS) reminders for adherence support (22). Data on MSWs is unavailable. Other service delivery characteristics that have been documented to influence decision-making around HIV prevention service uptake include waiting time, provider gender, provider attitude, availability of medical supplies, and distance to the service location. (21, 23, 24). Therefore, this study aimed to investigate the service delivery preferences for CAB-LA as an HIV PrEP option among heterosexual male and female sex workers in two urban cities in Uganda.**

3. In the methods section, it would be useful to explain why specific attributes such as location, waiting time, provider gender, and extra services were chosen for the discrete choice experiment

*Response: We apologize that we haven’t been very clear on why the specific attributes were chosen. However, in the methods under the subheading DCE attributes and level, we mention that a prior study identified location, waiting, provider gender, and extra services as key attributes influencing PrEP preferences. We also, we hypothesized that these service characteristics would similarly shape delivery preferences in this study (page 8, lines 5-10)*

**Based on existing literature, we identified several service delivery attributes that influence HIV service uptake, including service location, waiting time, provider gender, provider attitude, cost, distance to the service site, and availability of medical supplies (21, 23, 29-31). However, prior studies identified location, waiting time, provider gender, and the provision of additional services as key attributes influencing preferences for PrEP service delivery (32-35). Based on this evidence, we hypothesized that these service characteristics would similarly shape delivery preferences in this study**.

4. Further, the results would flow more clearly if they were organized into subsections, moving from participant characteristics to formulation preference and then to service delivery preferences.

*Response: We thank the reviewer for this suggestion. We had previously organized the results into subsections moving from characteristics of SWs, Condom use, syphilis/HIV testing behaviors, and PrEP use, and CAB-LA PrEP preferences. I have now changed to Participant characteristics of SWs, Condom use, syphilis/HIV testing behaviors, and PrEP preferences, and PrEP service delivery preferences as suggested (page 11, line 19; page 12, line 6; Page 12, line 20).*

5. An important omission is the frequency of CAB-LA, which should be explicitly stated as dosing every two months after an initial lead-in. In the interpretation, highlighting that the reduced dosing frequency helps explain why injectable PrEP is favored over oral PrEP would strengthen the discussion.

*Response: We thank the reviewer for the suggestion. We have adopted the suggestion and now a statement; CAB-LA is administered every two months following an initial loading dose, and this relatively infrequent schedule likely contributes to its preference over daily oral PrEP by reducing the adherence burden and supporting sustained protection has been included in the discussion (page 14, line 9)*

6. It would also be valuable to acknowledge the equity concern that private pharmacy or clinic delivery could exclude lower-income sex workers unless subsidized.

*Response: We thank the reviewer for the suggestion. We have adopted the recommendation and included a statement: Nonetheless, pharmacy-based delivery models may pose financial barriers, especially for SWs seeking integrated services or those with limited income, unless costs are subsidized (page 16, line 8)*

7. From a language and style perspective, shortening long sentences, reducing repetition of percentages, and improving readability would make the paper more polished.

Response: We thank the reviewer for the suggestion, and we have tried throughout the manuscript.

8. It is my opinion that the author adds some looking forward context about lenacapavir, a six-monthly injectable, in recommendations, probably to position the findings as forward-looking and relevant beyond CAB-LA.

*Response: We thank reviewer for the suggestion. We have now included the statement; Our findings highlight the importance of understanding user preferences for dosing schedules, delivery models, and supportive services, not only for CAB-LA but also for emerging six-monthly injectables such as lenacapavir. Implementation research and costing studies are needed to ensure feasibility, affordability, and sustainability (page 16, line 10)*

*9. Finally, the paper would benefit from a stronger closing statement emphasizing CAB-LA’s potential now and lenacapavir future promise, while stressing the need for sustainable, integrated, and equitable delivery models. This is to improve the relevance of this paper and make it forward-looking*

*Response; We thank the reviewer for this recommendation. The suggestion has been adopted, and the text below has been included (page 16, lines 17-21);*

**CAB-LA offers a promising opportunity to expand HIV prevention among SWs in Uganda, while emerging six-monthly injectables such as lenacapavir hold future potential to further enhance convenience, acceptability, and adherence. To maximize impact, implementation strategies must prioritize sustainable, integrated, and equitable delivery models that address user preferences, service accessibility, and adherence support for FSWs and MSWs.**

Reviewer #2:

1. All of my comments have been addressed. One last minor comment is that, while the participants were recruited from previously identified sex work hotspots and an established cohort of MSWs and FSWs, they were not all recruited from the established cohort. Therefore, I suggest that the author not use "cohort" to describe the participants in this study.

*Response: We thank the reviewer for the critical review of our work and grateful to the suggestion. We have replaced the word cohort in the description with study.*

We thank the reviewers for their helpful comments, which have improved the quality of our manuscript. We trust that the revised manuscript will be suitable for acceptance, but we are happy to consider further revisions if necessary.

Sincerely

Richard Muhindo, PhD, MPH, BSN

**Senior Lecturer**, Department of Nursing, College of Health Sciences

Makerere University

Corresponding author

Email: r.muhindo@yahoo.com
